# Blood soluble interleukin 1 receptor accessory protein levels are consistently low throughout the menstrual cycle of women with endometriosis

**DOI:** 10.1186/1477-7827-12-51

**Published:** 2014-06-16

**Authors:** Nadège Michaud, Mahera Al-Akoum, Ali Akoum

**Affiliations:** 1 Centre Hospitalier Universitaire de Québec, Hôpital Saint-François d’Assise, 10, rue de l’Espinay, Québec D0-711, Canada

**Keywords:** Endometriosis, Inflammation, IL1, IL1RAP

## Abstract

**Background:**

A deficiency in the counter-regulatory mechanisms of interleukin 1 (IL1) may play a significant role in endometriosis pathogenesis and associated chronic inflammation. The aim of this study was to investigate peripheral blood levels of soluble IL1 receptor accessory protein (sIL1RAP), a potent natural inhibitor of IL1, in women with and without endometriosis.

**Methods:**

Peripheral blood samples were collected from women with endometriosis (n = 47) consulting for infertility, pelvic pain or tubal ligation, in whom the disease was diagnosed at laparoscopy. Control healthy women (n = 27) were requesting tubal ligation or reanastomosis and had no visible evidence of endometriosis at laparoscopy. sIL1RAP levels were determined by ELISA, whereas estradiol (E2) and progesterone (P4) levels were determined by competitive immunoassays.

**Results:**

sIL1RAP levels were significantly decreased in women with early endometriosis stages compared to controls (p < 0.05) and markedly during the proliferative phase of the menstrual cycle (p < 0.001). Actually, while sIL1RAP were significantly increased in the proliferative compared to the secretory phase in normal women (p < 0.0001) and peaked at the end of this phase, sIL1RAP remained consistently low and showed non-significant variations throughout the menstrual cycle in women with endometriosis.

**Conclusions:**

Lower circulating levels of sIL1RAP points to a significant impairment in the counter-regulatory mechanisms of IL1, which in view of the cytokine’s potent inflammatory and growth-promoting properties may play a significant role in the pathophysiology of endometriosis.

## Background

Endometriosis is one of the most frequent gynaecological diseases in reproductive age women. A one in ten woman suffers from endometriosis in North America. Defined as an ectopic growth of endometrial tissue, endometriosis is frequently associated pelvic pain and infertility (30-40%) [1] and a chronic pelvic inflammation [2].

Cumulative evidence clearly points to a significant role for immune factors in endometriosis development. Inflammation is a major hallmark of endometriosis, but the underlying mechanisms are not clearly elucidated. Local immune surveillance appears to be deficient in women with endometriosis and immune cells seem to paradoxically favor ectopic endometrial cell growth *via* different mechanisms that include secretion of inflammatory, angiogenic and growth mediators such as monocyte chemotactic protein 1 (MCP1), macrophage migration inhibitory factor (MIF), interleukin 1 (IL1), IL8, and vascular endothelial cell growth factor (VEGF) [3]. These contribute to endometriosis-associated chronic peritoneal inflammation, but also to endometrial cell survival, attachment to the peritoneal tissue, invasion, proliferation and activation of the host angiogenic responses [3]. Endometriotic implants are likely to stimulate leukocyte recruitment and activation, and endometrial dysfunctions may contribute to the activation of peritoneal immune cells and exacerbate the inflammatory reaction *via* cyclical reflux of menstrual debris into the peritoneal cavity [3-7].

Nevertheless, alterations in immune functions observed in patients having endometriosis are not restricted to the peritoneal cavity, where the disease is frequently found. Systemic alterations including increased levels of cytokines [8], reduced cytotoxic effects of lymphocytes on autologous endometrial cells and increased activation of peripheral blood monocytes [9,10] were reported. These cells also appeared to stimulate endometrial cell growth *in vitro*[9,11].

Our and other previous studies found reduced concentrations of sIL1R2 in the peripheral blood of women with endometriosis [12,13]. This decoy receptor is a potent natural inhibitor of IL1 [14], which suggests an impairment in IL1 surveillance and counter-regulatory mechanisms in women with endometriosis at the systemic level. IL1 activates cells *via* its receptor type 1 (IL1R1) and the involvement of membrane-bound receptor accessory protein (mIL1RAP) [15]. However, a soluble form of IL1RAP (sIL1RAP), which results from alternative splicing rather than form proteolytic cleavage of the membrane-bound receptor extracellular domain, has been described. sIL1RAP was detected in human serum and appeared to play an important role in the counter-regulation of IL1 effects by increasing the affinity of IL1 binding to sIL1R2 [16].

Our subsequent studies showed that sIL1RAP levels were significantly decreased in the endometrium and peritoneal fluid of women with endometriosis [17,18]. The aim of this study was then to evaluate sIL1RAP concentrations in sera of endometriosis patients and investigate the influence of endometriosis stage and main symptoms (pain and infertility) and the menstrual cycle phase

## Methods

### Subjects

Women were recruited into the study after providing an informed consent for a research protocol approved by the CHU de Quebec Ethics Committee on Human Research. Subjects with endometriosis (n = 47; mean age, 31.9 ± 4.3 years) were consulting for pelvic pain, dysmenorrhea, dyspareunia and/or infertility or were asymptomatic and requesting tubal ligation. Endometriosis was diagnosed at laparoscopy/laparotomy before any medical or surgical intervention and staged according to the revised classification of the American Fertility Society (rAFS) [19]. Women with endometriosis who were included in this study displayed no other pelvic pathology and had not taken any anti-inflammatory or hormone medication at least three months before laparoscopy. Control subjects (n = 27; mean age, 36.2 ± 5.6 years) were fertile women undergoing laparoscopy for tubal ligation or reanastomosis in whom neither laparoscopic evidence of endometriosis nor other pathologies (leiomyoma, uterine fibroids or pelvic infection) are found. These women were fertile, had regular menstrual cycles, and had not taken anti-inflammatory or hormone medication at least three months before laparoscopy. The menstrual cycle phase (proliferative or secretory) for all patients was determined based on cycle history, serum oestradiol (E_2_) and progesterone (P4) levels and histological examination of endometrial biopsies when available according to the criteria of Noyes et al. [20]. The main clinical characteristics used in this study are summarized in Table 1.

**Table 1:** Clinical characteristics of patients at the time of laparoscopy

	**No. of patients**	**Age (yr) (mean ± SD)**
***Controls***	27	36.2 ± 5.6
***Endometriosis***	47	31.9 ± 4.3
*Stages I-II*	36	32.1 ± 4.6
*Stages III-IV*	11	31.0 ± 2.8
*Fertile*	11	33.0 ± 5.2
*Infertile*	36	31.7 ± 4.0
*Without pain*	33	31.8 ± 4.4
*With pain*	14	31.9 ± 3.8
***Proliferative phase***		
*Controls*	14	34.3 ± 6.4
*Endometriosis*	19	30.9 ± 4.5
***Secretory phase***		
*Controls*	13	37.8 ± 4.7
*Endometriosis*	28	32.5 ± 4.0

### Collection and processing of blood samples

Blood was collected in sterile tubes containing ethylenediaminetetracetic acid (EDTA), immediately centrifuged at 2,000 *g* for 10 minutes at 4°C, and the serum aliquoted and stored at −80°C until assay. For hormonal assays, blood was collected in redtop tubes and sent to the biochemistry laboratory for current steroid determination.

### IL1RAP enzyme-linked immunosorbent assay (ELISA)

Soluble IL1RAP concentrations in sera were measured by an ELISA procedure designed in our laboratory. Ninety-six-well microtiter plates (Corning Incorporated Life Sciences, Lowell, MA, USA) were coated overnight at 4°C with anti-hIL1RAP polyclonal antibody [0.71 μg/mL in phosphate buffered saline (PBS)] (R&D Systems, Minneapolis, MN, USA). Wells were washed with PBS containing 0.1% Tween-20 (PBS-Tween) and incubated for 1 h at 37°C with PBS containing 3% bovine serum albumin (BSA) (PBS-BSA). Samples and shIL1RAP (R&D Systems) were diluted in PBS-BSA, added to wells and incubated successively with biotinylated anti-shIL1RAP polyclonal antibody (0.01 μg/mL in PBS-BSA) (R&D Systems) for 1 h at 37°C, peroxidase-labeled streptavidin HRP-peroxidase (1 μg/mL in PBS-BSA) (Jackson ImmunoResearch Laboratories, West Grove, PA) for 45 min at 37°C and finally with TMB (3,3′, 5,5′,-tetramethylbenzidine) (Bio-Rad Laboratories Ltd, Mississauga, Ontario, Canada) as peroxidase substrate for 5 min at room temperature. The reaction was then stopped with 2 N sulphuric acid (H_2_SO_4_) and the optical density (OD) determined at 450 nm. sIL1RAP concentrations were calculated by interpolation from standard curves using shIL1RAP (R&D Systems) at concentrations varying between 2 and 128 ng/mL and showing a sensitivity limit of approximately 0.4 ng/mL. Measurements were performed in duplicate. All samples were assessed at the same time to prevent any possible changes due to freezing and thawing. The inter- and intra-assay coefficients of variation for the sIL1RAP assay were 5.8% and 8.0%, respectively.

### E_2_ and P4 assays

Serum E_2_ and P4 were measured by competitive immunoassays based on antibody-coated tubes (commercial kits, Coat-A-Count; Diagnostic Products Corp., Los Angeles, CA). The intra-assay coefficients of variation measured at low, medium, and high levels of the standard curves were between 1.8% and 8.0% for all the immunoassays. The inter-assay coefficients of variation were less than 8.0% for E_2_ and 10% for P4.

### Statistical analysis

sIL1RAP concentrations followed a Gaussian distribution and were statistically analysed using one-way analysis of variance (ANOVA) and the Bonferroni’s test for *post hoc* for multiple comparisons. Comparison of 2 groups was performed using the unpaired t test (Prism 3.0, GraphPad Software, San Diego, CA, USA). Differences were considered as statistically significant at p < 0.05. The statistical power was calculated online using a Statistical Power Calculator with a two-sided α level of 0.05 (https://www.dssresearch.com/KnowledgeCenter/toolkitcalculators/statisticalpowercalculators).

## Results

**Figure 1:**
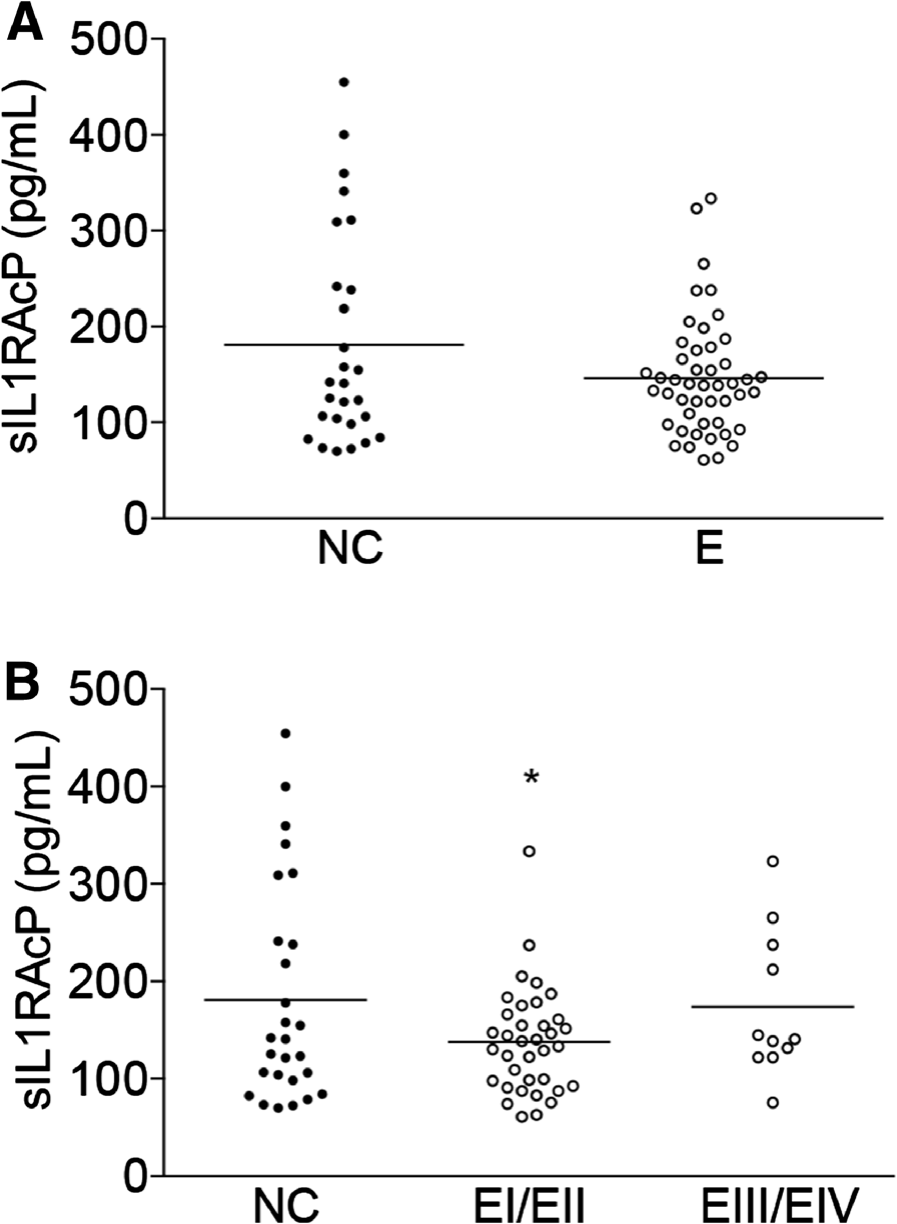
**sIL1RAP concentrations in blood samples of patients without or with different endometriosis stages. A**, normal controls (NC) and women with endometriosis (E); **B**, normal controls (NC) and women with endometriosis distributed according to the stage of the disease (EI/II and EIII/IV). The horizontal lines represent the mean for each set of data. *p < 0.05 as compared to NC.

**Figure 2:**
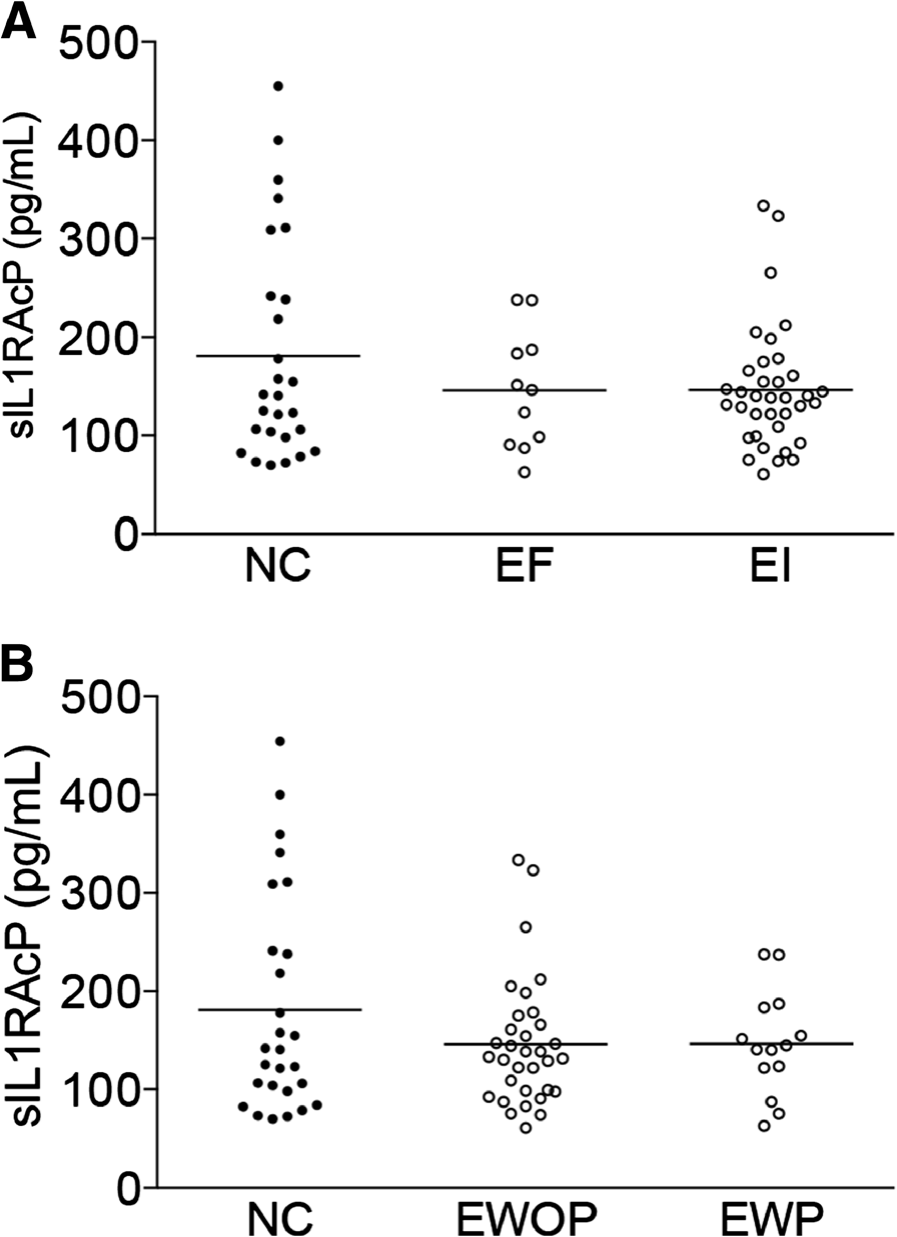
**sIL1RAP concentrations in blood samples of patients without or with endometriosis distributed according to the fertility status or pelvic pain. A**, normal controls (NC) and women with endometriosis distributed according to the fertility status (EF, endometriosis fertile and EI, endometriosis infertile); **B**, normal controls (NC) and women with endometriosis distributed according to the absence or presence of pelvic pain (EWOP, endometriosis without pain and EWP, endometriosis with pain). The horizontal lines represent the mean for each set of data.

sIL1RAP was detected in the peripheral blood and the distribution of its concentrations in normal controls and women with endometriosis are presented in Figure 1. sIL1RAP levels were slightly decreased in women with endometriosis, but this decrease did not reach the level of statistical significance (p = 0.08) (Figure 1A). A significant decrease was nevertheless observed in women with endometriosis stages I-II compared to normal controls (p < 0.05), but not in the advanced stages III-IV of the disease (Figure 1B). Data analysis taking into account the fertility status of women with endometriosis or the presence and absence of pelvic pain showed no statistically significant differences with normal controls (Figure 2A and B).

**Figure 3:**
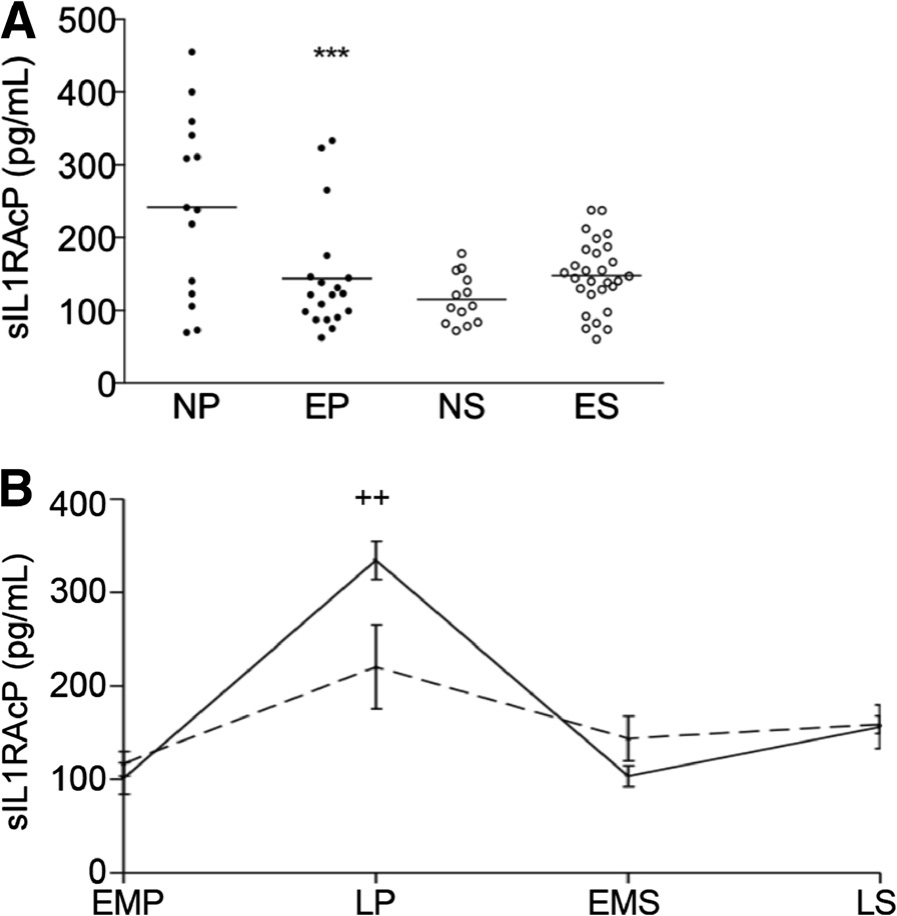
**sIL1RAP concentrations in blood samples of patients without and with endometriosis distributed according to the menstrual cycle phase. A**, sIL1RAP concentration in normal controls and women with endometriosis in the proliferative (NP and EP, respectively) and the secretory (NS and ES, respectively) phases of the cycle. **B**, Cyclic variations of sIL1RAP concentrations in women with and without endometriosis. EMP, early-mid proliferative; LP, late proliferative; EMS, early-mid secretory; LS, late secretory. The horizontal lines represent the mean for each set of data; ***p < 0.001 as compared to NP; ++p < 0.01 as compared to NC.

Analysis of sIL1RAP levels according to the menstrual cycle phase first showed a significant diminution during the secretory phase compared to the proliferative phase in normal controls (p < 0.0001) (statistical power, 97.5%) (Figure 3A). In women with endometriosis, no significant difference between the proliferative and the secretory phases was noted. As compared to normal controls however, patients with endometriosis showed a statistically significant diminution of sIL1RAP levels in the proliferative phase (p < 0.001), but not in the secretory phase (statistical power, 100% and 95%, respectively) (Figure 3A). Further distribution of sIL1RAP values along the menstrual cycle showed a peak, which, in normal women, was reached at the end of the proliferation phase. In women with endometriosis, however, the amplitude of such a peak was significantly lower (p < 0.01) compared to normal controls (statistical power, 80%), and no significant cycle-dependent changes could be observed (Figure 3B). Since these results suggest a hormonal regulation of sIL1RAP levels, we then analysed E_2_ and P4 concentrations in the same serum samples. Data displayed in Table 2 showed no statistically significant differences in E_2_ or P4 levels between normal controls and women with endometriosis in the proliferative or the secretory phase of the menstrual cycle. Furthermore, no significant correlation between sIL1RAP levels and those of E_2_ or P4 was found in women with endometriosis or normal controls (data not shown).

**Table 2:** E2 and P4 levels in sera of patients

	**No. of patients**	**E2 (pmol/L) (mean ± SEM)**	**P4 (nmol/L) (mean ± SEM)**
***Follicular phase***			
*Controls*	14	300.8 ± 65.4	5.8 ± 2.4
*Endometriosis*	19	268.3 ± 39.6	2.3 ± 0.3
***Luteal phase***			
*Controls*	13	375.7 ± 74.1	17.6 ± 4.1
*Endometriosis*	28	485.1 ± 57.6	26. 2 ± 3.8

## Discussion

In the present study, we found that sIL1RAP levels were not significantly altered in the peripheral blood of women with endometriosis; only a slight diminution was observed compared to normal women. Our data did not point to any noticeable relationship with endometriosis-related infertility or pelvic pain, as sIL1RAP levels were comparable in fertile and infertile women with endometriosis and in those who have or not pelvic pain. However, data analysis according to endometriosis stage revealed a statistically significant decrease in the earliest stages of the disease. The number of EIII/IV patients included in this study was too small to draw a definite conclusion on sIL1RAP relationship with advanced endometriosis stages. Nevertheless, our findings are consistent with the available literature showing that endometriosis is biochemically more active in the early stages. Actually, it is quite believed that endometriotic lesion types represent distinctive steps in the evolutionary process of the disease and that initial and highly active, vascularized and inflammatory red lesions display an increased expression of pro-inflammatory, angiogenic and tissue remodelling factors such as nuclear factor (NF)-kB, IL1R1, MIF, vascular endothelial cell growth factor (VEGF) and prostaglandins (PGs) [21-25].

Analysis of sIL1RAP concentrations according to the menstrual cycle further revealed a significant decrease during the proliferative phase in women with endometriosis compared to controls, and precisely at the end of this phase; a phenomenon that was not observed during the secretory phase. Actually, sIL1RAP levels were significantly lower in the proliferative than in the secretory phase in normal healthy controls, whereas in women with endometriosis they showed no significant cycle-dependent variations and remained low throughout the menstrual cycle. Analysis of E_2_ and P4 levels in the peripheral blood sera of the same women did not reveal any significant endometriosis-related changes, nor did it identify a significant correlation with sIL1RAP levels, which rules out their involvement in the regulation of sIL1RAP levels in the peripheral blood. The decrease in circulating sIL1RAP levels remains unexplained and so are the source(s) of sIL1RAP secretion. sIL1RAP was detected in human serum [16], but it is unknown whether immune blood cells, the predominant cell population in the peripheral blood, could be responsible for sIL1RAP production. Furthermore, immune cell dysfunctions have been described in sera of patients with endometriosis and could explain this phenomenon [3,7-9,26,27]. However, we cannot completely rule out an eventual endometrial origin since our previous study showed that human endometrium is capable of producing sIL1RAP [18]. Considering that the endometrium is a highly vascularized tissue, a decrease in the secretion of endometrial sIL1R3 could, at least partly, explain our current results. It would however be interesting to evaluate the production of sIL1R3 in immune blood cells of women with and without endometriosis.

Our findings may have a significant relevance for endometriosis pathophysiology. Actually, sIL1RAP is a major specific inhibitor of IL1. Unlike the constitutive membrane-bound form of IL1RAP, which is necessary for IL1-mediatd cell activation *via* the functional activating IL1R1, sIL1RAP potentiates IL1 affinity for the decoy inhibitory IL1R2 and plays thereby a significant role in the down-regulation of the cytokine’s pro-inflammatory effects [14,16,28]. IL1 A and B forms were detected in the peripheral blood, but their increased concentrations in women with endometriosis reported by some studies were not corroborated by other studies [12,13,29]. However, the significant decline in serum sIL1RAP levels in endometriosis stages I-II shown in the current study together with the reduced concentration of sIL1R2 shown by our and other previous studies, particularly in stages I-II as well [12,13], clearly point to a significant deficiency in the specific regulatory mechanisms of IL1 at the systemic level in the earliest stages of the disease. Endometriosis-associated immune and inflammatory changes were mainly located within the peritoneal cavity of endometriosis patients, where the disease frequently develops, and especially within endometriotic lesions where immune cell infiltration and endometriotic cell activation and expression of inflammatory, tissue remodelling and growth factors were reported [2,8,30-32]. However, although relatively less obvious, intrauterine and even systemic immune-inflammatory changes, such as increased activation of monocytes and secretion of pro-inflammatory cytokines, have also been observed in women with endometriosis [3,7-9,26,27].

sIL1RAP was found to be down-regulated in eutopic endometrial tissue of women with endometriosis and a significant decline in the concentration of this molecule was found in the peritoneal fluid as well [33]. This, considering the pro-inflammatory, angiogenic and growth promoting properties of IL1 [14,34-36] and its increased production in endometriotic tissue [33], suggests that a combination of local and systemic deficiency in sIL1RAP may lead to an increased endometriotic cell activation by IL1 and contribute to the promotion of the ectopic growth and development of these cells. It is noteworthy that because of its potent anti-inflammatory properties, IL1RAcP has been shown to ameliorate collagen-induced arthritis *in vivo* using a collagen-induced-arthritis mouse model [28,37]. In fact, its ability to interact with IL1 seems to play a substantial role in the regulation of inflammation since it has been approved for therapeutic use as an IL1 trap fusion protein [38]. Therefore, it would be of interest to evaluate sIL1RAcP efficacy as a potential treatment of endometriosis using mouse models.

## Conclusions

Our study showed for the first time a cycle phase dependency for sIL1RAP levels in the peripheral blood of normal healthy women, which peak in the proliferative phase of the menstrual cycle, but instead showed non-significant variations throughout the menstrual of women with endometriosis. Lower circulating levels of sIL1RAP may, in combination a deficiency in sIL1RAP expression in eutopic endometrium and a reduction in its peritoneal fluid concentrations, be relevant to endometriosis pathophysiology as it points to a significant lack in the counter-regulatory mechanisms of IL1-mediated inflammation, tissue remodelling and cell growth. The source(s) of sIL1RAP deficiency in the peripheral blood and the involved regulatory pathways need to be investigated.

## Competing interests

The authors declare that they have no competing interests.

## Authors’ contributions

NM performed the experiments and wrote the first version of the manuscript. MA contributed to statistical analysis and helped in the analysis of the results and writing of the manuscript. AA set up design, supervised experiments and data analysis and reviewed, corrected and finalized the manuscript. All authors have read and approved the final manuscript.
